# ﻿Morpho-phylogenetic evidence reveals novel species and new records of *Torula* (Torulaceae, Pleosporales) from medicinal plants in China

**DOI:** 10.3897/mycokeys.122.161816

**Published:** 2025-09-11

**Authors:** Na Wu, Mei-Feng Chi, Hong-Zhi Du, K. W. Thilini Chethana, Kitiphong Khongphinitbunjong, Ya-Ya Chen, Kevin D. Hyde, Fatimah Al-Otibi, Asha J. Dissanayake, Jian-Kui Liu

**Affiliations:** 1 The Clinical Hospital of Chengdu Brain Science Institute, School of Life Science and Technology, University of Electronic Science and Technology of China, Chengdu 611731, China; 2 Center of Excellence in Fungal Research, Mae Fah Luang University, Chiang Rai 57100, Thailand; 3 School of Science, Mae Fah Luang University, Chiang Rai 57100, Thailand; 4 School of Pharmacy, Guizhou University of Traditional Chinese Medicine, Guiyang 550025, Guizhou Province, China; 5 Department of Entomology and Plant Pathology, Faculty of Agriculture, Chiang Mai University, Chiang Mai 50200, Thailand; 6 Institute of Crop Germplasm Resources, Guizhou Academy of Agricultural Sciences, Guiyang 550006, China; 7 Department of Botany and Microbiology, College of Science, King Saud University, P.O. Box 22452, Riyadh 11495, Saudi Arabia

**Keywords:** Asexual morph, Dothideomycetes, multi-locus, taxonomy, two new taxa

## Abstract

During a saprobic fungi survey in China, five hyphomycetous fungi of *Torula* (Torulaceae, Pleosporales, Dothideomycetes) were discovered. These taxa were characterized and identified based on morphology, culture characteristics, and phylogenetic analysis of the combined ITS, LSU, SSU, *tef*1-α, and *rpb*2 sequence dataset. Consequently, two novel species, *Torula dispora* and *T. strychnicola*, are introduced based on the distinct spinulose characteristics on the conidial substrate and the size of conidia. Phylogenetic results indicated that our isolates of *T. dispora* and *T. strychnicola* each represent a distinct lineage. Additionally, three new host records of *T. chinensis*, *T. fici*, and *T. masonii* are reported. Based on morphological and phylogenetic evidence, we formally propose the synonymization of *T. phytolaccae* under *T. chinensis*. Detailed descriptions, illustrations, and informative notes are provided for all species.

## ﻿Introduction

Medicinal plants, as a valuable resource for modern drug discovery and development—such as anticancer, antibacterial, and antiviral medications—play a significant role in human healthcare (Anand et al. 2019; Ali et al. 2021; Atanasov et al. 2021; Adeleye et al. 2022; Chaachouay and Zidane 2024). The growth and reproduction, as well as the quality, of these medicinal herbs are influenced by numerous factors. It is worth noting that microfungi are a crucial factor affecting the quality of medicinal plants, including endophytes (Keshri et al. 2021; Purushotham et al. 2021; Zhang et al. 2024a), pathogens (Niu et al. 2016; Petrasch et al. 2019), and saprobes (Medina et al. 2020; Yang et al. 2022; Sun et al. 2025). Given the wide variety and rich resources of medicinal plants (Chen et al. 2016; Rasool et al. 2020), they provide a suitable habitat for exploring and discovering fungal diversity.

Torulaceae was initially established by Sturm (1829) to accommodate the type genus *Torula*, which is only known by its asexual morph. Crous et al. (2015) confirmed its familial placement within Pleosporales and accepted two genera, *Dendryphion* and *Torula*, in the family based on multilocus phylogeny and morphology (Su et al. 2016, 2018; Li et al. 2017; Boonmee et al. 2021). To date, seven genera have been accepted in Torulaceae according to the latest outline by Hyde et al. (2024). This family is characterized by septate, subcylindrical conidiophores with or without apical branches; doliiform to ellipsoid or clavate, smooth to verruculose, mono- to polyblastic conidiogenous cells; and subcylindrical or fusiform, smooth to verrucose conidia arranged in branched chains (Crous et al. 2014, 2015; Hyde et al. 2016; Li et al. 2016; Su et al. 2016, 2018). Members of Torulaceae are distributed worldwide, and most taxa are saprobic, found on dead or decaying wood in freshwater and terrestrial habitats (Crous et al. 2015; Su et al. 2016, 2018; Li et al. 2017; Pratibha and Prabhugaonkar 2017; Hyde et al. 2020; Boonmee et al. 2021).

*Torula* was initially introduced by Persoon (1794) and is typified by *T. herbarum*, which was accepted within Torulaceae by Crous et al. (2015) based on multilocus phylogenetic analysis. This genus is primarily characterized by cupulate conidiogenous cells produced in branched chains, as well as subglobose, verrucose, septate conidia (Crous et al. 2015; Crane and Miller 2016; Hyde et al. 2016; Su et al. 2018). Species of *Torula* are found on a wide range of hosts and are widely distributed in both terrestrial and freshwater habitats worldwide (Su et al. 2018; Hyde et al. 2020; Boonmee et al. 2021). In recent years, the number of species of *Torula* has gradually increased with the continuous discovery of new species and records (Li et al. 2023; Wang et al. 2023; Dong et al. 2023; He et al. 2024; Tian et al. 2024; Zhang et al. 2024b). However, studies on species from this genus occurring on medicinal plants are limited.

During an investigation of hyphomycetous fungi in terrestrial habitats, seven isolates representing five species of *Torula* were identified from five different medicinal plants that had not been previously studied in China. Morphological comparisons coupled with a multilocus phylogeny revealed two novel species and three new host records of *Torula* from these new collections.

## ﻿Materials and methods

### ﻿Sample collection, examination, and isolation

In this study, dead specimen materials were collected from medicinal plants in Guizhou, Hainan, and Sichuan provinces, China. These samples were stored in envelopes and brought to the laboratory. Morphological observations of fungal structures were made using a Nikon SMZ745 dissecting microscope (Nikon Corporation, Tokyo, Japan). Photomicrographs of the fungal specimens were captured using a Nikon Eclipse Ni-U compound microscope fitted with a Nikon DS-Ri2 digital camera (Nikon Corporation, Tokyo, Japan). Macromorphological structures were photographed with a Nikon SMZ800N stereo microscope fitted with a Nikon DS-Fi3 microscope camera (Nikon Corporation, Tokyo, Japan). All measurements were made using the Tarosoft Image Frame Work program v. 0.97, and the photo plates were prepared with Adobe Photoshop CC extended version 21.1.2. Single-spore isolations were conducted using the methods described by Senanayake et al. (2020). Germinated spores were transferred to fresh PDA plates and incubated under dark conditions at 25 °C. Colony characteristics were observed and recorded after 2 weeks, following the method described by Rayner (1970).

Dry plant specimens were deposited in the Herbarium of Cryptogams, Kunming Institute of Botany, Chinese Academy of Sciences (HKAS), Kunming, China; the Herbarium of University of Electronic Science and Technology (HUEST), Chengdu, China; and the Guizhou Academy of Agriculture Sciences (GZAAS), Guiyang, China. Pure cultures were deposited in the CGMCC (CGMCC), Beijing, China; the University of Electronic Science and Technology Culture Collection (UESTCC), Chengdu, China; and the Guizhou Culture Collection (GZCC), Guizhou, China. Faces of fungi and Index Fungorum numbers were obtained for the new taxa as in Jayasiri et al. (2015) and Index Fungorum (www.indexfungorum.org), respectively.

### ﻿DNA extraction, PCR amplification, and sequencing

Fresh mycelia (about 50–100 mg) were scraped using a sterilized toothpick from the margin of a colony on the PDA plate, which was incubated under dark conditions at 25 °C for 2–3 weeks (Wu et al. 2001), and stored in 1.5 mL sterilized microcentrifuge tubes. The TreliefTM Plant Genomic DNA Kit (TSINGKE Biotech, Shanghai, P.R. China) was used to extract DNA according to the manufacturer’s instructions. The obtained genomic DNA was stored in two tubes, one at 4 °C for polymerase chain reaction (PCR) amplification and the other at -20 °C for long-term storage. The internal transcribed spacer region of rDNA (ITS), nuclear large subunit rDNA (28S, LSU), nuclear small subunit rDNA (18S, SSU), RNA polymerase second-largest subunit (*rpb*2), and translation elongation factor 1-alpha (*tef*1-α) were selected for the study. The amplifications were performed in a 25 μL reaction volume containing 9.5 μL ddH2O, 12.5 μL 2× Taq PCR Master Mix with blue dye (Sangon Biotech, Shanghai, P.R. China), 1 μL of DNA template, and 1 μL of each primer. PCR product purification and sequencing were performed at Beijing Tsingke Biotechnology (Chengdu) Co., Ltd., China. The loci, primers, and amplification conditions used are listed in Table 1.

**Table 1.** Loci, primers, and amplification conditions used in this study.

Locus	Primers	Optimized PCR Protocols	References
ITS	ITS5	94 °C 3 min; 36 cycles of 94 °C 45 s, 55 °C 55 s, 72 °C 1 min; 10 min at 72 °C; 4 °C on hold	White et al. (1990)
	ITS4		
LSU	LR0R	94 °C 3 min; 36 cycles of 94 °C 45 s, 55 °C 55 s, 72 °C 1 min; 10 min at 72 °C; 4 °C on hold	Vilgalys and Hester (1990); Rehner and Samuels (1994)
	LR5		
SSU	NS1	94 °C 3 min; 36 cycles of 94 °C 45 s, 55 °C 55 s, 72 °C 1 min; 10 min at 72 °C; 4 °C on hold	White et al. (1990)
	NS4		
*rpb*2	fRPB2-5F	95 °C 5 min; 40 cycles of 95 °C 15 s, 56 °C 50 s, 72 °C 2 min; 10 min at 72 °C; 4 °C on hold	Liu et al. (1999)
	fRPB2-7cR		
*tef*1-α	EF1-983F	94 °C 3 min; 38 cycles of 94 °C 45 s, 56 °C 55 s, 72 °C 1 min; 10 min at 72 °C; 4 °C on hold	Rehner and Buckley (2005); Rehner (2001)
	EF1-2218R		

### ﻿Phylogenetic analysis

Sequences generated in this study were checked and assembled using BioEdit v.17.0.1 (Hall 2006) to ensure sequence quality. Using the BLASTn search tool on NCBI (https://blast.ncbi.nlm.nih.gov/Blast.cgi), based on newly generated ITS and LSU sequence data, we found that our taxa were similar to species of Torulaceae. According to the BLAST results and previous literature, appropriate sequences were downloaded from GenBank to construct a phylogenetic tree. Two isolates of *Cylindrotorula indica* (ex-type strain NFCCI 4836 and NFCCI 4837) were selected as outgroup taxa. Details of the sequences used in this study are listed in Table 2. The sequences were aligned using MAFFT v.7 online (https://mafft.cbrc.jp/alignment/server/) and AliView (Larsson 2014). The alignments were trimmed using trimAl v.1.2 (Capella-Gutierrez et al. 2009) with minimal coverage (-cons = 0.8) and gap threshold (-gt = 0.6), and the results were checked in BioEdit v.17.0.1 (Hall 2006). Concatenation of the loci was conducted using SequenceMatrix v.1.8 (Vaidya et al. 2011). The Nexus and Phylip files for phylogenetic analysis were generated in AliView (Larsson 2014). Phylogenetic analysis of the combined sequence data was performed using maximum likelihood (ML) and Bayesian inference (BI) methods, as detailed by Dissanayake et al. (2020). The ML analysis was carried out with RAxML-HPC2 v.8 on XSEDE (8.2.12) through the CIPRES Science Gateway v.3.3 (https://www.phylo.org/portal2/login!input.action) (Silvestro and Michalak 2012). The tree search included 1,000 nonparametric bootstrap replicates; the best-scoring tree was selected among suboptimal trees from each run by comparing likelihood scores under the GTRGAMMA substitution model. The BI analysis was conducted in MrBayes v.3.2.6 (Ronquist et al. 2012). MrModeltest v.2.3 (Nylander 2008) was used to determine the best nucleotide substitution model for each data partition. The GTR+I+G model was selected for ITS, LSU, and *tef*1-α regions; the GTR+I model for SS; and the SYM+I+G model for the *rpb*2 region.

**Table 2.** GenBank accession numbers and details of the isolates chosen for the phylogenetic study.

Taxa	Strain/Specimen number	GenBank Accession Number					References
		ITS	LSU	SSU	*tef*1-*α*	*rpb*2	
*Cylindrotorula indica*	NFCCI 4837	MT339445	MT339443	N/A	MT321493	MT321491	Boonmee et al. (2021)
*C. indica*	NFCCI 4836 ^T^	MT339444	MT339442	N/A	MT321492	MT321490	Boonmee et al. (2021)
*Torula acaciae*	CBS 142113 ^T^	NR155944	NG059764	N/A	N/A	KY173594	Crous et al. (2016)
*T. aquatica*	KUMCC 15-0435 ^T^	MG208167	MG208146	N/A	N/A	MG207977	Su et al. (2018)
*T. aquilariae*	KUNCC 24-18640 ^T^	PQ788522	PQ788524	N/A	PQ810572	PQ810570	Li et al. (2025)
*T. breviconidiophora*	KUMCC 18-0130 ^T^	MK071670	MK071672	MK071697	MK077673	N/A	Hyde et al. (2019)
*T. calceiformis*	KUNCC 22-12449 ^T^	OP751054	OP751052	OP751050	OQ630512	OQ630510	Hyde et al. (2023)
*T. camporesii*	KUMCC 19-0112 ^T^	MN507400	MN507402	MN507401	MN507403	MN507404	Hyde et al. (2020)
*T. canangae*	MFLUCC 21-0169 ^T^	OL966950	OL830816	N/A	ON032379	N/A	de Silva et al. (2022)
*T. chiangmaiensis*	KUMCC 16-0039 ^T^	MN061342	KY197856	KY197863	KY197876	N/A	Li et al. (2017)
*T. chinensis*	UESTCC 22.0085 ^T^	OQ127986	OQ128004	OQ127995	N/A	N/A	Tian et al. (2023)
***T. chinensis* (*T. phytolaccae*)**	**ZHKUCC 22-0107**	**ON611796**	**ON611800**	**ON611798**	**ON660881**	**ON660879**	**Li et al. (2023)**
***T. chinensis* (*T. phytolaccae*)**	**ZHKUCC 23-0884**	**OR365458**	**OR365488**	**OR365493**	**OR700205**	**N/A**	**Dong et al. (2023)**
***T. chinensis***	**UESTCC 24.0234**	**PV577741**	**PV577746**	**PV569592**	**PV706476**	**PV706481**	**In this study**
*T. chromolaenae*	KUMCC 16-0036 ^T^	MN061345	KY197860	KY197867	KY197880	KY197873	Li et al. (2017)
*T. dingjieensis*	HKAS 144529 ^T^	PQ684995	PQ675414	PQ675375	PQ671467	PQ671471	He et al. (2025)
***T. dispora***	**CGMCC 3.27461** ^T^	**PQ394056**	**N/A**	**N/A**	**PQ346496**	**PQ379949**	**In this study**
***T. dispora***	**UESTCC 23.0503**	**PQ394057**	**N/A**	**N/A**	**PQ346497**	**PQ379950**	**In this study**
*T. fici*	CBS 595.96 ^T^	KF443408	KF443385	KF443387	KF443402	KF443395	Crous et al. (2015)
*T. fici*	KUMCC 16-0038	MN061341	KY197859	KY197866	KY197879	KY197872	Li et al. (2017)
*T. fici*	KUMCC 15-0428	MG208172	MG208151	N/A	MG207999	MG207981	Su et al. (2018)
*T. fici*	UESTCC 22.0124	OQ127979	OQ127997	OQ127988	N/A	OQ158970	Tian et al. (2023)
***T. fici***	**UESTCC 24.0235**	**PV577742**	**PV577747**	**PV569593**	**PV706477**	**PV706482**	**In this study**
*T. gaodangensis*	MFLUCC 17-0234 ^T^	MF034135	NG 059827	NG 063641	N/A	N/A	Hyde et al. (2020)
*T. goaensis*	NFCCI 4040 ^T^	NR 159045	NG 060016	N/A	N/A	N/A	Pratibha and Prabhugaonkar (2017)
*T. guizhouensis*	KUNCC 23-13912 ^T^	PQ671230	PQ671150	N/A	PQ662575	PQ662502	Zhang et al. (2025)
*T. herbarum*	CBS 140066 ^T^	KR873260	KR873288	N/A	N/A	N/A	Crous et al. (2015)
*T. hollandica*	CBS 220.69 ^T^	NR 132893	NG 064274	KF443389	KF443401	KF443393	Crous et al. (2015)
*T. hydei*	KUMCC 16-0037 ^T^	MN061346	MH253926	MH253928	MH253930	N/A	Li et al. (2020)
*T. kanvae*	MCC-10010 ^T^	PQ248154	PQ248155	PQ248153	PQ287916	PQ284948	Crous et al. (2024)
*T. lancangjiangensis*	DLUCC 2043 ^T^	MW723059	MW879526	MW774582	MW729785	MW729780	Boonmee et al. (2021)
*T. lancangjiangensis*	MFLUCC 21-0099	MZ538529	MZ538563	N/A	MZ567104	N/A	Boonmee et al. (2021)
*T. longan*	ZHKUCC 22-0121 ^T^	OR194035	OR194027	OR194032	OR228537	OR228535	Dong et al. (2023)
*T. longan*	ZHKUCC 22-0122	OR194036	OR194028	OR194033	OR228538	OR228536	Dong et al. (2023)
*T. luguhuensis*	KUNCC 22-12427 ^T^	OQ729758	OQ947766	N/A	OQ999004	OQ999002	Luan et al. (2023)
*T. mackenziei*	MFLUCC 13-0839 ^T^	MN061344	KY197861	KY197868	KY197881	KY197874	Li et al. (2017)
*T. masonii*	CBS 245.57 ^T^	NR 145193	NG 058185	N/A	N/A	N/A	Crous et al. (2015)
***T. masonii***	**GZCC25-0020**	**PV577743**	**PV577748**	**PV569594**	**PV706478**	**N/A**	**In this study**
*T. pluriseptata*	KUMCC 16-0034 ^T^	MN061338	KY197855	KY197862	KY197875	KY197869	Li et al. (2017)
*T. polyseptata*	KUMCC 18-0131 ^T^	MK071671	MK071673	MK071698	MK077674	N/A	Hyde et al. (2019)
*T. sichuanensis*	UESTCC 22.0087 ^T^	OQ127981	OQ127999	OQ127990	N/A	N/A	Tian et al. (2023)
***T. strychnicola***	**GZCC 24-0163** ^T^	**PV577744**	**PV577749**	**N/A**	**PV706479**	**PV706483**	**In this study**
***T. strychnicola***	**GZCC 24-0146**	**PV577745**	**PV577750**	**N/A**	**PV706480**	**PV706484**	**In this study**
*T.* sp.	CBS 246.57	KF443411	KR873290	N/A	N/A	N/A	Crous et al. (2015)
*T. suae*	CGMCC 3.24259 ^T^	OP359406	OP359415	OP369300	OP471618	OP476730	Wang et al. (2023)
*T. submersa*	UESTCC 22.0086 ^T^	OQ127985	OQ128003	OQ127994	N/A	OQ158968	Tian et al. (2023)
*T. sundara*	MFLUCC 21-0067	OM276824	OM287866	N/A	N/A	N/A	Jayawardena et al. (2022)
*T. thailandica*	GZCC 20-0011 ^T^	MN907426	MN907428	MN907427	N/A	N/A	de Silva et al. (2022)
*T. woodwardiae*	KUNCC 23-13941 ^T^	PQ671232	PQ671152	N/A	PQ662577	PQ662504	Zhang et al. (2025)
*T. yadongensis*	HKAS 144533 ^T^	PQ684997	PQ675416	PQ675377	PQ671469	PQ671473	He et al. (2025)

* **Remarks**: The superscript T denotes ex-type isolates. “N/A” denotes the sequence is unavailable. The newly generated sequences, new species, and synonymized isolates are indicated in black bold font. **Abbreviations: CBS**: CBS−KNAW Fungal Biodiversity Center, Utrecht, The Netherlands; **CGMCC**: China General Microbiological Culture Collection Center, Institute of Microbiology, Chinese Academy of Sciences, Beijing, China; **DLUCC**: Dali University Culture Collection, Yunnan, China; **GZCC**: Guizhou Culture Collection, Guizhou, China; **HKAS**: Herbarium of Cryptogams, Kunming Institute of Botany, Chinese Academy of Sciences, China; **KUMCC**: Kunming Institute of Botany Culture Collection, Yunnan, China; **KUNCC**: Kunming Institute of Botany Culture Collection Center, Kunming, China; **MCC**: Microbial Culture Collection, India; **MFLUCC**: Mae Fah Luang University Culture Collection, Chiang Rai, Thailand; **NFCCI**: National Fungal Culture Collection of India, India; **UESTCC**: University of Electronic Science and Technology Culture Collection, Chengdu, China; **ZHKUCC**: Zhongkai University of Agriculture and Engineering Culture Collection, Guangzhou, China.

Phylogenetic trees were visualized with FigTree v.1.4.0 (http://tree.bio.ed.ac.uk/software/figtree/) and further edited in Adobe Illustrator 2020 (Adobe Systems Inc., USA). The final alignment was submitted to Figshare (https://figshare.com) at https://doi.org/10.6084/m9.figshare.26085910 (accessed on 10 July 2025).

## ﻿Results

### ﻿Phylogenetic analysis

**Figure 1.**
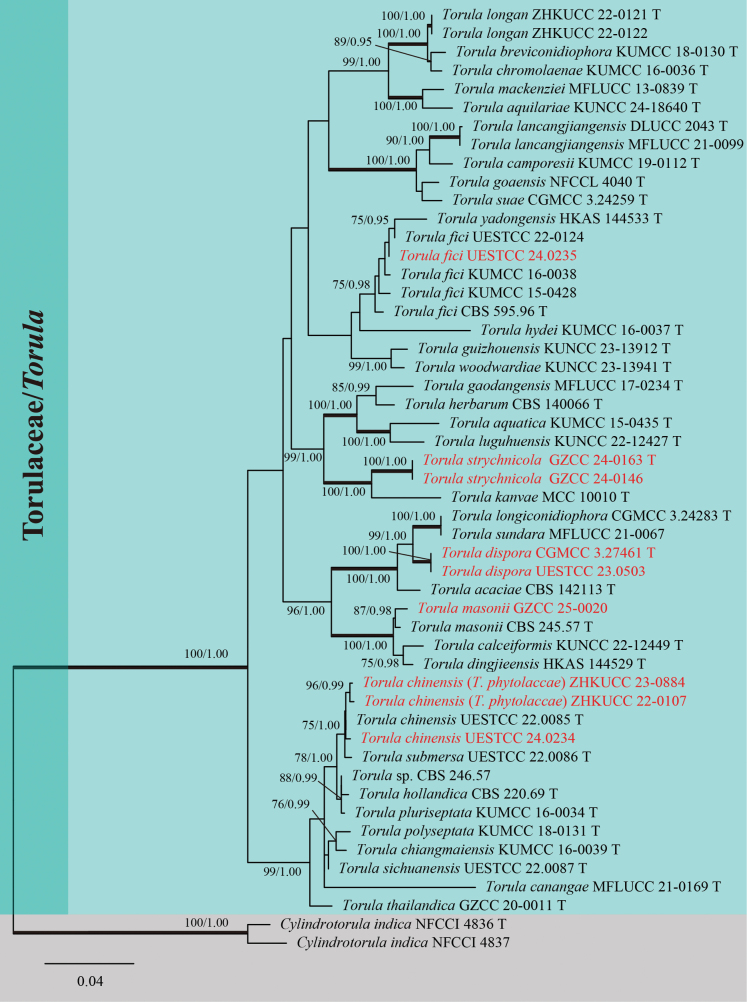
Phylogenetic tree constructed from maximum likelihood (RAxML) analysis based on the combined ITS, LSU, SSU, *tef*1-α, and *rpb*2 sequence data for selected members of *Torula* (Torulaceae). Branch support for ML equal to or greater than 75% and Bayesian inference posterior probabilities (BIPP) equal to or greater than 0.95 are marked above or below branches as MLBS/BIPP. The abbreviation ‘T’ indicates the ex-type strain. Species names and culture collections in red are newly collected taxa and synonymized isolates. The tree was rooted with *Cylindrotorula indica* (ex-type strains NFCCI 4836 and NFCCI 4837).

The analyzed dataset was composed of the combined ITS, LSU, SSU, *tef*1-α, and *rpb*2 sequence data of 49 ingroup taxa and two outgroup taxa of *Cylindrotorula indica* (ex-type strains NFCCI 4836 and NFCCI 4837) (Fig. 1). The aligned dataset comprised 4,177 characters (ITS: 496 bp, LSU: 841 bp, SSU: 996 bp, *tef*1-α: 883 bp, and *rpb*2: 961 bp), including gaps. The RAxML analysis of the combined dataset yielded a best-scoring tree (Fig. 1) with a final ML optimization likelihood value of -17,726.033710. The matrix had 1,082 distinct alignment patterns, with 27.10% undetermined characters or gaps. Estimated base frequencies were A = 0.245460, C = 0.263536, G = 0.269505, and T = 0.221499; substitution rates were AC = 1.547028, AG = 3.458151, AT = 1.360383, CG = 0.894323, CT = 7.519490, and GT = 1.000000; and the gamma distribution shape parameter (alpha) = 0.120960. The tree length was 1.092604. The ML and BI phylogenetic analyses resulted in trees with similar topologies. Tree topology was consistent with previous studies by Dong et al. (2023) and Tian et al. (2023).

In this study, the multilocus phylogenetic analysis showed that our seven isolates were nested within the family Torulaceae. UESTCC 24.0235, UESTCC 24.0234, and GZCC 25-0020 clustered with *Torula fici* (ex-type strain CBS 595.96, KUMCC 16-0038, KUMCC 15-0428, and UESTCC 22.0124), *T. chinensis* (UESTCC 22.0085, ex-type strain), and *T. masonii* (CBS 245.57, ex-type strain), respectively. Two isolates (CGMCC 3.27461 and UESTCC 23.0503) of *Torula dispora* were sister to *T. sundara* (MFLUCC 21-0067) and *T. longiconidiophora* (CGMCC 3.24283, ex-type strain) and formed a monophyletic lineage with strong support (99% MLBS/1.00 BIPP). Two isolates (GZCC 24-0163 and GZCC 24-0146) of *Torula strychnicola* were sister to *T. kanvae* (MCC-10010, ex-type strain) with strong support (100% MLBS/1.00 BIPP) and formed a distinct lineage. In addition, *T. phytolaccae* (ZHKUCC 22-0107 and ZHKUCC 23-0884) and our collection (UESTCC 24.0234) clustered with *T. chinensis* (UESTCC 22.0085, ex-type strain).

### ﻿Taxonomy

#### ﻿ Torula chinensis

Taxon classificationFungiPleosporalesTorulaceae

W.H. Tian, Y.P. Chen & Maharachch., J. Fungi 9 (2, no. 150): 4 (2023)

DA385709-EADA-5F48-9F4A-3A1EDE32B56F

847014

Fig. 2

= Torula phytolaccae Y.X. Li, C.F. Liao & Doilom, Phytotaxa 584(1): 9 (2023)

##### Description.

***Saprobic*** on dead branches of *Phytolacca americana* L. (Phytolaccaceae). **Sexual morph**: Undetermined. **Asexual morph**: Hyphomycetous. ***Colonies*** effuse on the natural substrate, dense, velvety, scattered, hairy, dark brown to black, dry. ***Mycelium*** immersed to superficial on the substrate, septate, branched, smooth to minutely verruculose, brown. ***Conidiophores*** 10–28 × 3–6 μm (*x̄* = 20 × 5 μm, n = 20), micronematous to semi-macronematous, mononematous, flexuous, unbranched, verruculose, thick-walled, doliiform to subcylindrical, consisting of 1–3 cells or reduced to conidiogenous cells, smooth, straight, or slightly flexuous, pale brown to brown. ***Conidiogenous cells*** 4–7 × 5–8 μm (*x̄* = 5 × 5.5 μm, n = 20), mono- or polyblastic, integrated, lateral to terminal, dark brown to black, verruculose, thick-walled, doliiform to cupulate. ***Conidia*** (16–) 22–35 (–65) × 5–9 μm (*x̄* = 29 × 7 μm, n = 30), solitary to catenate, acrogenous, simple, phragmosporous, straight or slightly curved, dark brown to black, verrucose, predominantly 3–5-septate, rounded at both ends, mostly subcylindrical, composed of subglobose cells, thick-walled, constricted at the septa, subhyaline to pale brown at the apex.

**Figure 2.**
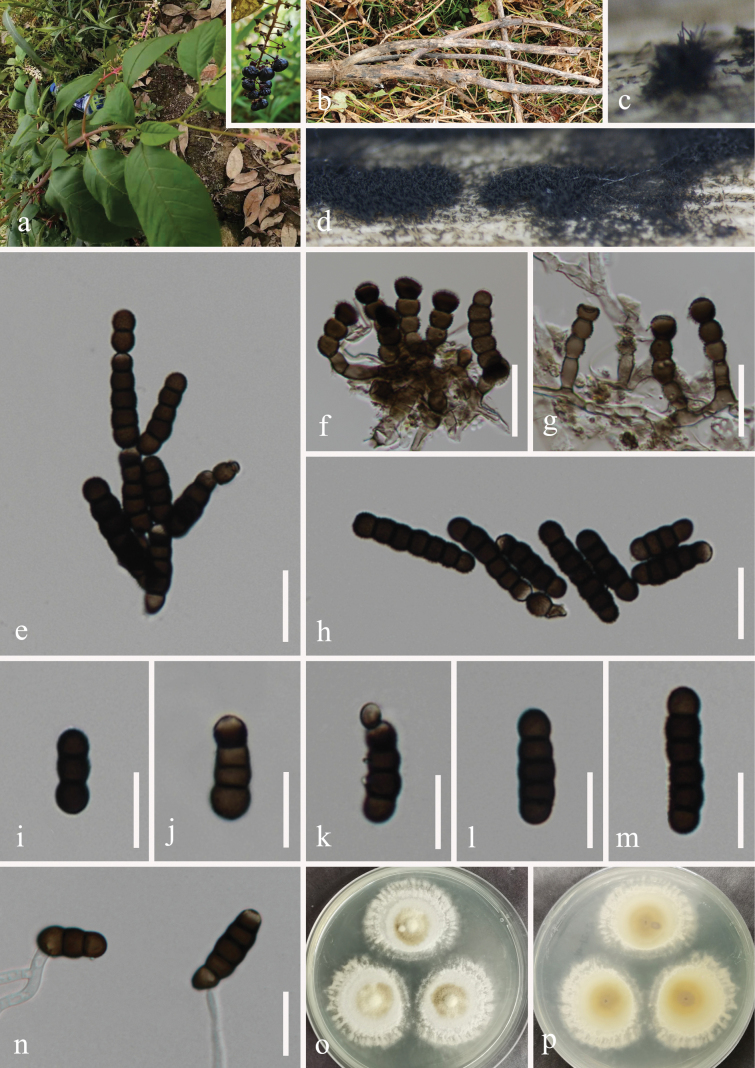
*Torula chinensis* (HUEST 24.0247, new host record). a. Host *Phytolacca americana*; b. Branches of *P. americana*; c, d. Colonies on the host surface; e, h−m. Conidia; f, g. Conidiophores, conidiogenous cells, and conidia; n. Germinated conidium; o, p. Colonies on PDA, above (o) and below (p). Scale bars: 50 μm (e, f); 20 μm (g); 15 μm (h−n).

##### Culture characteristics.

Conidia germinated on the PDA within 24 h, and germ tubes are produced from the basal cells. Colonies growing on PDA reached 30–33 mm in diam. after three weeks at 25 °C in the dark. Colonies from above are medium dense, regular, white aerial mycelium slightly raised, fluffy, filiform, pale greenish-grey in the center, white hairy at the margin; in reverse, pale yellow at the center, off-white at the margin, the color gradually lightens from the center to the edge, without pigment produced in PDA.

##### Material examined.

China • Guizhou Province, Guiyang City, Huaxi District, 26°30'41"N, 106°39'23"E, elevation 1,126 m, on dead branches of *Phytolacca americana* (Phytolaccaceae), 24 January 2021, Hong-Zhi Du, S62 (HUEST 24.0247), living culture UESTCC 24.0234.

##### Notes.

*Torula chinensis* was introduced by Tian et al. (2023) from unidentified dead woody substrates and formally published on 22 January 2023. Around the same time, *T. phytolaccae* was described by Li et al. (2023) from dead stems of *Phytolacca acinosa* and published on 9 February 2023. In our phylogenetic tree (Fig. 1), our collection (UESTCC 24.0234) and *T. phytolaccae* (ZHKUCC 22-0107 and ZHKUCC 23-0884) clustered together with the ex-type strain (UESTCC 22.0085) of *T. chinensis*. Comparisons of nucleotides between *T. chinensis* (UESTCC 22.0085) and *T. phytolaccae* (ZHKUCC 22-0107, ex-type strain) showed 100% similarity (534/534 bp, without gaps) in ITS, 99.9% (803/804 bp, without gaps) in LSU, and 99.4% (810/815 bp, without gaps) in SSU. Additionally, our collection (UESTCC 24.0234) shows 100% similarity to *T. chinensis* in the ITS, LSU, and SSU gene regions. Therefore, following the guidelines of Jeewon and Hyde (2016), we synonymized *T. phytolaccae* under *T. chinensis*, identified our collection as *T. chinensis*, and reported it as a new host record from the medicinal plant *Phytolacca americana* in Guizhou Province, China.

#### ﻿ Torula dispora

Taxon classificationFungiPleosporalesTorulaceae

H.Z. Du, N. Wu, K.D. Hyde & Jian K. Liu sp. nov.

1C1C951E-A88C-5EE4-A740-6CE072CB640B

855436

Fig. 3

##### Etymology.

The epithet ‘*dispora*’ refers to the host genus *Disporum* from which the fungus was originally isolated.

##### Holotype.

HKAS 132495

##### Description.

***Saprobic*** on dead twigs of *Disporum cantoniense* (Loureiro) Merrill (Liliaceae). **Sexual morph**: Undetermined. **Asexual morph**: Hyphomycetous. ***Colonies*** effuse on the natural substrate, scattered, hairy, dark brown to black, dry. ***Mycelium*** mostly immersed, hyaline, septate, branched hyphae. ***Conidiophores*** 15–40 × 4–6 μm (*x̄* = 32 × 5 μm, n = 20), micronematous to semi-macronematous, subcylindrical, erect, septate, smooth, straight, or slightly flexuous, brown to dark brown. ***Conidiogenous cells*** 5–8 × 4–5 μm (*x̄* = 7 × 4.5 μm, n = 20), monoblastic, integrated, terminal, subglobose or spherical to coronal, brown to dark brown. ***Conidia*** two types; short *conidia* 16–35 × 5–9 μm (*x̄* = 27 × 7 μm, n = 30), acrogenous, phragmosporous, branched in chains with subglobose cells, dry, brown to dark brown, subhyaline at the terminal cell, 4–9-septate, constricted at septa, and the central cells larger than end cells, verrucose, easily separating, with spinulose on the substrate; long *conidia* 178–389 × 4–6 μm (*x̄* = 256 × 5 μm, n = 30), acrogenous, phragmosporous, dry, straight to flexuous, pale brown to brown, terminal cell subhyaline, 20–40-septate, constricted at septa, verrucose, easily separating, fusiform to ellipsoidal cells and uniform in size.

**Figure 3.**
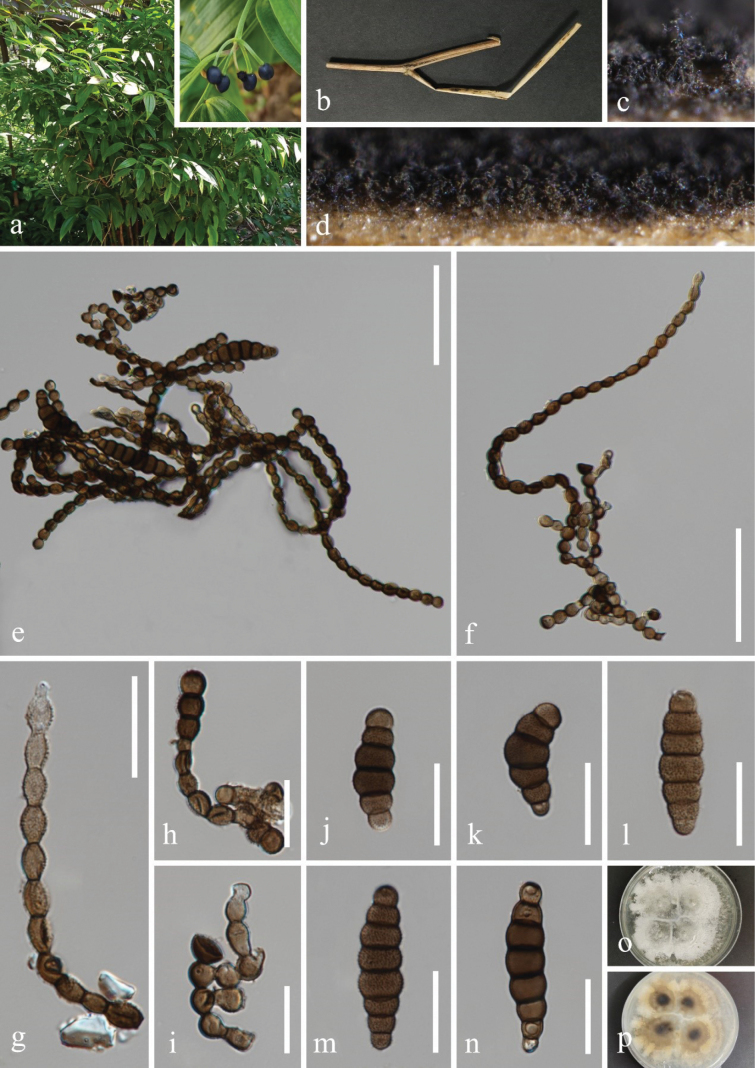
*Torula dispora* (HKAS 132495, holotype). a. Host *Disporum cantoniense*; b. Twigs of *D. cantoniense*; c, d. Colonies on the host surface; e−h. Conidiophores with conidia; i Conidiophores with conidiogenous cells; j−n. Conidia; o, p. Colonies on PDA, above (o) and below (p). Scale bars: 50 μm (e, f); 20 μm (g); 15 μm (h−n).

##### Culture characteristics.

Conidia germinated on the PDA within 24 h, and germ tubes are produced from the basal cell. Colonies growing on PDA reached 45–48 mm in diam. after three weeks at 25 °C in the dark. Colonies from above irregular, mycelium slightly raised, fluffy, filiform, white aerial hyphae at the surface, spreading from the center, and hyaline mycelium at the edge; in reverse, brown to dark-brown in the center, with grayish white at the edge, the color gradually lightens from the center to the edge.

##### Material examined.

China • Sichuan Province, Chengdu City, Dujiangyan City, Qingcheng Mountain scenic spot, 30°55'9"N, 103°28'36"E, elevation 1,185 m, on dead twigs of *Disporum cantoniense* (Liliaceae), 27 March 2021, H.Z. Du, S173 (HKAS 132495, holotype), ex-type living culture CGMCC 3.27461; • ibid., S82 (HUEST 23.0504), living culture UESTCC 23.0503.

##### Notes.

Multi-locus phylogeny (Fig. 1) showed that *Torula dispora* (CGMCC 3.27461 and UESTCC 23.0503) clustered together with *T. longiconidiophora* (CGMCC 3.24283) and *T. sundara* (MFLUCC 21-0067) with high support (99% MLBS/1.00 BIPP), forming a distinct lineage. Morphologically, *T. dispora* (HKAS 132495, holotype) is similar to *T. longiconidiophora* and *T. sundara* in having micronematous to semi-macronematous conidiophores, terminal, subglobose or spherical to coronal conidiogenous cells, and two types of conidia (short and long) forming in chains (Jayawardena et al. 2022; Tian et al. 2023). However, *T. dispora* (HKAS 132495, holotype) can be distinguished from *T. longiconidiophora* and *T. sundara* by the spinulose characteristics on the conidial substrate (Jayawardena et al. 2022; Tian et al. 2023). In addition, *T. dispora* differs from *T. sundara* by its smaller short-type conidia (16–35 × 5–9 μm vs. 41–60 × 9–15 μm) and fewer septa in conidia (20–40 vs. up to 50) (Jayawardena et al. 2022). The nucleotide base pair comparison between *T. dispora* (CGMCC 3.27461, ex-type strain) and *T. sundara* (KUNCC 22-12399) revealed 10/509 bp (2.0%, 2 gaps) in ITS, 16/900 bp (1.8%, 4 gaps) in *tef*1-α, and 24/885 bp (2.7%, without gaps) in *rpb*2 differences (He et al. 2024), while *T. dispora* (CGMCC 3.27461) can be distinguished from *T. longiconidiophora* (CGMCC 3.24283, ex-type strain) by 11/519 bp (2.1%, 3 gaps) in ITS and 33/982 bp (3.3%, without gaps) in *rpb*2 sequence data (Tian et al. 2023). Therefore, based on the morphological and phylogenetic evidence (Jeewon and Hyde 2016), *T. dispora*, associated with the medicinal plant *Disporum cantoniense*, is introduced as a new species from Sichuan Province, China.

#### ﻿ Torula fici

Taxon classificationFungiPleosporalesTorulaceae

Crous [as ‘ ficus’], IMA Fungus 6 (1): 192 (2015)

A843FAF5-676E-5130-96D3-94995D4AAF3A

816154

Fig. 4

##### Description.

***Saprobic*** on the dead vines of *Lonicera japonica* Thunb. (Caprifoliaceae). **Sexual morph**: Undetermined. **Asexual morph**: Hyphomycetous. ***Colonies*** effuse on the natural substrate, scattered, black, clustered on substrates. ***Mycelium*** slightly immersed, septate, branched, smooth, with pale brown hyphae. ***Conidiophores*** arising from prostrate hypha, 4–6 μm wide, macronematous to semi-macronematous, mononematous, solitary, erect, thick-walled, erect, straight, or slightly flexuous, without apical branches, pale brown to brown, ellipsoid to subcylindrical, smooth, septate. ***Conidiogenous cells*** 5.5–6.5 × 4.8–5.5 μm (*x̄* = 6 × 5 μm, n = 20), mono- to polyblastic, integrated, terminal, thick-walled, doliiform to subglobose, brown to dark brown. ***Conidia*** 8–19 × 5–7 μm (*x̄* = 15 × 6 μm, n = 30) phragmosporous, solitary to catenate, acrogenous, pale brown, aseptate or indistinctly septate at immature stage, becoming brown to dark brown at maturity, composed of subglobose cells, apical cell often pale brown, rounded at both ends, mostly 2–4-septate, constricted at the septa, minutely verruculose, easily separating.

**Figure 4.**
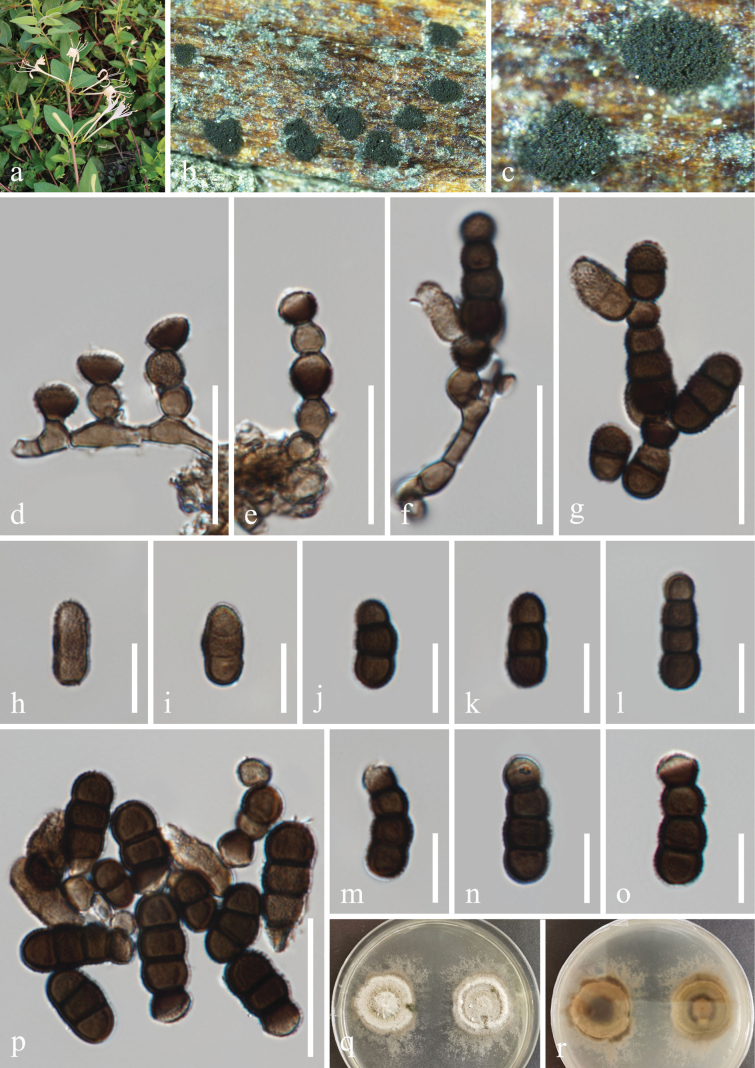
*Torula fici* (HUEST 24.0248, new host record). a. Host *Lonicera japonica*; b, c. Colonies on the host surface; d−f. Conidiophores and conidiogenous cells; g−o. Conidia; q, r. Colonies on PDA, above (q) and below (r). Scale bars: 20 μm (d−g, p); 10 μm (h−o).

##### Culture characteristics.

Conidia germinated on the PDA within 24 h, and germ tubes are produced from the basal cell. Colonies growing on PDA reached 35–40 mm in diam. after three weeks at 25 °C in the dark, mycelium partly immersed to superficial, slightly effuse, hairy, with a regular edge, pink or pale brown. Colonies from above, circular with an irregular edge, white aerial mycelium slightly raised, medium dense, fluffy, filiform, spreading from the center, and hyaline mycelium at the edge; in reverse, pale brown to brown in the center, with off-white at the edge.

##### Material examined.

China • Sichuan Province, Chengdu City, Xindu District, Xinfan Street, 30°50'51"N, 104°4'49"E, elevation 549 m, on dead vines of *Lonicera japonica* (Caprifoliaceae), 22 July 2021, H.Z. Du, D10 (HUEST 24.0248), living culture UESTCC 24.0235.

##### Notes.

*Torula fici* was introduced by Crous et al. (2015) as a hyphomycetous asexual morph species found on *Ficus religiosa* in Cuba. It is a widespread species, mostly reported in China and Thailand, and has been collected from various hosts, including *Ananas comosus*, *Chromolaena odorata*, *Cocos nucifera*, *Garcinia* sp., *Magnolia grandiflora*, *Mangifera indica*, *Musa* sp., and *Pandanus* sp. (Jayasiri et al. 2019; Jayawardena et al. 2022; Tian et al. 2024; Zhang et al. 2024b). Based on the phylogenetic analysis (Fig. 1), our collection (UESTCC 24.0235) clustered with *T. fici* (CBS 595.96, KUMCC 16-0038, KUMCC 15-0428, and UESTCC 22.0124). The morphology of our collection matches well with the type material of *T. fici*, which is characterized by doliiform to subglobose or clavate conidiogenous cells and brown to dark brown, phragmosporous, verruculose, branched chains and septate conidia (Crous et al. 2015). Therefore, we identified our new collection as *T. fici* and reported its association with the medicinal plant *Lonicera japonica* in Sichuan Province, China, for the first time.

#### ﻿ Torula masonii

Taxon classificationFungiPleosporalesTorulaceae

P.W. Crous, IMA Fungus 6 (1): 192 (2015)

51B782E9-FDFB-54FA-AB70-DB23C38648CE

812806

Fig. 5

##### Description.

***Saprobic*** on dead branches of *Gardenia jasminoides* J. Ellis (Rubiaceae). **Sexual morph**: Undetermined. **Asexual morph**: Hyphomycetous. ***Colonies*** effuse on the natural substrate, scattered, hairy, velvety, dark brown to black. ***Mycelium*** immersed or superficial, composed of septate, branched, dark brown to black hyphae. ***Conidiophores*** 2–4 µm wide, macronematous to semi-macronematous, septate, erect, straight or slightly flexuous, brown to dark brown, subcylindrical to subglobose, thick-walled, with 1–2 doliiform to globose cells. ***Conidiogenous cells*** 6–9 × 5–8 µm (*x̄* = 7.5 × 6.5 μm, n = 20), polyblastic, terminal or intercalary, dark brown to black, doliiform to subglobose, smooth to verruculose, thick-walled. ***Conidia*** 20–60 × 4–9 μm (*x̄* = 36 × 6 μm, n = 30), phragmosporous, catenated, acrogenous, minutely verruculose, rounded at both ends, composed of subglobose cells, dark brown to black, 1–2 smaller cells at the apex, 3–18-septate, easily separating, slightly constricted at some septa, and chiefly subcylindrical.

**Figure 5.**
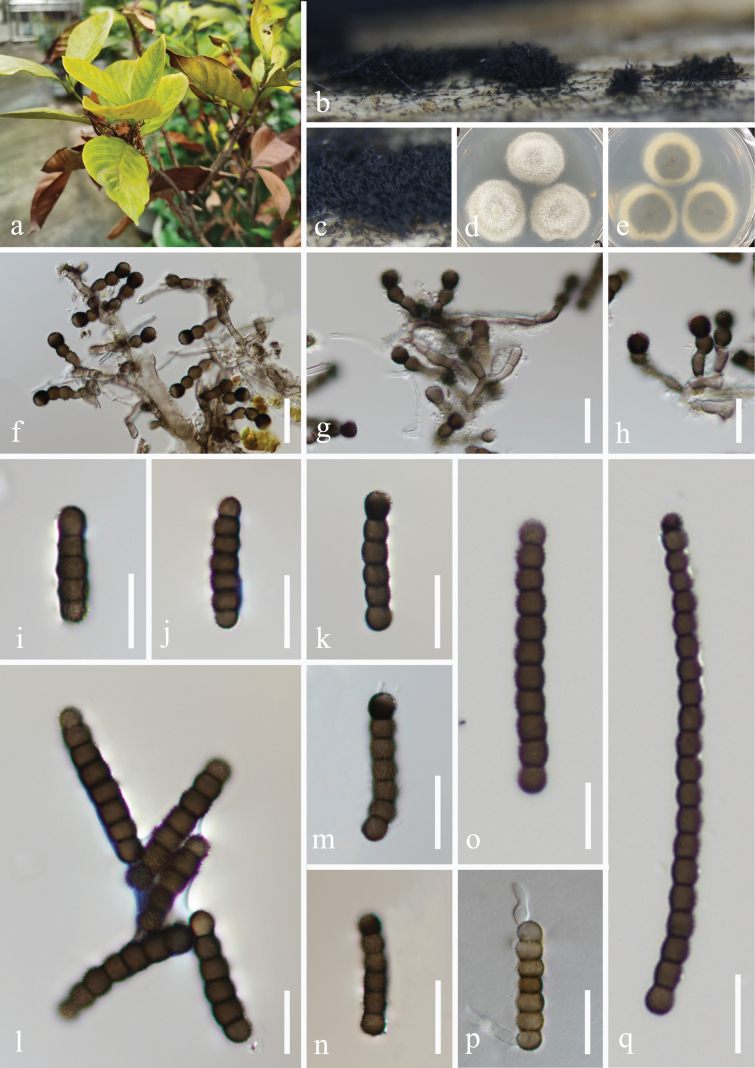
*Torula masonii* (GZAAS 25-0020, new host record). a. Host *Gardenia jasminoides*; b, c. Colonies on the host surface; d, e. Colonies on PDA, above (d) and below (e); f−h. Conidiophores and conidiogenous cells; i−o, q. Conidia; p. Germinated conidium. Scale bars: 20 μm (f−h); 15 μm (i−n, p); 10 μm (o, q).

##### Culture characteristics.

Conidia germinated on the PDA within 24 h, and germ tubes are produced from the basal cell. Colonies growing on PDA reached 33–35 mm in diam. after three weeks at 25 °C in the dark. Colonies from above medium dense, regular, white aerial mycelium slightly raised, fluffy, filiform. In reverse, greenish-grey in the center, off-white ring at the margin, without pigments produced in PDA.

##### Material examined.

China • Sichuan Province, Guangyuan City, Qingquan Town, 32°41'35"N, 105°58'39"E, elevation 817 m, on dead branches of *Gardenia jasminoides* (Rubiaceae). 20 April 2021, H.Z. Du, L2092 (GZAAS 25-0020), living culture GZCC 25-0020.

##### Notes.

Crous et al. (2015) introduced *Torula masonii*, which was collected from *Brassica* sp. in the UK. Subsequently, Li et al. (2017) and Su et al. (2018) recollected and described *T. masonii* from *Iris germanica* in Italy and submerged decaying wood in China, respectively. In this study, multi-locus phylogenetic analysis showed that our isolate (GZCC 25-0020) clustered together with *T. masonii* ex-type (CBS 245.57) with 87% MLBS and 0.98 BIPP support (Fig. 1). Additionally, our collection (GZAAS 25-0020) shows similar morphological characteristics to *T. masonii* (CBS H-22278). Therefore, we identified our new collection as *T. masonii*, which is associated with the medicinal plant *Gardenia jasminoides* in Sichuan Province, China.

#### ﻿ Torula strychnicola

Taxon classificationFungiPleosporalesTorulaceae

H.Z. Du, N. Wu, K.D. Hyde & Jian K. Liu sp. nov.

DFB4D396-74D7-52D3-87DE-ADB1E26242EB

856185

Fig. 6

##### Etymology.

The epithet ‘*strychnicola*’ refers to the host genus *Strychnos* from which the fungus was originally isolated.

##### Holotype.

HKAS 139505

##### Description.

***Saprobic*** on dead branches of medicinal plant *Strychnos nux-vomica* L. (Loganiaceae). **Sexual morph**: Undetermined. **Asexual morph**: Hyphomycetous. ***Colonies*** effuse on the natural substrate, sparse, hairy, velvety, dark brown to black on the substrate. Mycelium immersed or superficial, composed of septate, branched, dark brown to black hyphae. ***Conidiophores*** 2–4 µm wide, macronematous to semi-macronematous, erect, septate, smooth, straight, or slightly flexuous, brown to dark brown, subcylindrical to subglobose, and thick-walled, with 1–2 doliiform to globose cells. ***Conidiogenous cells*** 4–6 × 3–5 μm (*x̄* = 5 × 4 μm, n = 20), monoblastic, integrated, terminal, subglobose or spherical, brown to dark brown. ***Conidia*** (16–) 25–50 (–65) × 5–8 μm (*x̄* = 38 × 7 μm, n = 30), catenated, acrogenous, simple, phragmosporous, arranged in branched chains, dry, brown to dark brown, cylindrical, rounded at both ends, composed of globose to ellipsoidal cells, occasionally smaller at the apex (1–2 cells black at the apex), subhyaline at the terminal cell, 2–15-septate, constricted at septa, verrucose, with spinulose on the substrate.

**Figure 6.**
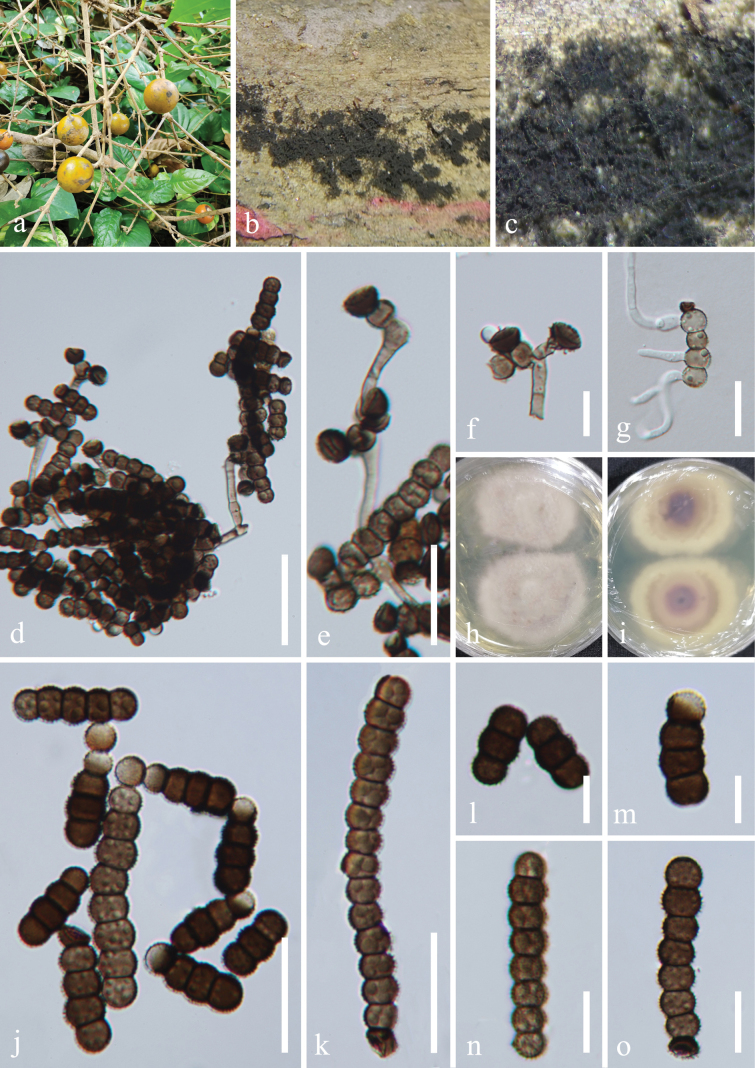
*Torula strychnicola* (HKAS 139505, holotype). a. Host *Strychnos nux-vomica*; b, c. Colonies on the host surface; d, e. Conidiophores, conidiogenous cells with conidia; f. Conidiophores with conidiogenous cells; g. Germinated conidium; h, i. Colonies on PDA, above (h), below (i); j−o. Conidia. Scale bars: 30 μm (d); 20 μm (e); 10 μm (f); 20 μm (g, j, k); 10 μm (l−o).

##### Culture characteristics.

Conidia germinated on the PDA within 24 h, and germ tubes are produced from the basal cell. Colonies growing on PDA reached 40–43 mm in diam. after two weeks at 25 °C in the dark. Colonies from above regular, white aerial mycelium slightly raised, medium dense, fluffy, filiform, velvety, pale purple; in reverse, purplish brown at the center, with greyish white at the edge, with pale brown ring between them, the color gradually lightens from the center to the margin.

##### Material examined.

China • Hainan Province, Wuzhishan City, Shuiman River tropical rainforest scenic area in Wuzhishan, 18°53'29"N, 109°34'36"E, elevation 1,845 m, on dead branches of *Strychnos nux-vomica* (Loganiaceae), 15 August 2021, Jian Ma, WZS71.1 (HKAS 139505, holotype), ex-type living culture GZCC 24-0163; • ibid., WZS73 (GZAAS 23-0630, isotype), living culture GZCC 24-0146.

##### Notes.

Multi-locus phylogeny (Fig. 1) showed that *Torula strychnicola* (GZCC 24-0163 and GZCC 24-0146) was sister to *T. kanvae* (MCC-10010) with strong support (100% MLBS/1.00 BIPP) and formed a distinct lineage. Morphologically, *Torula strychnicola* can be distinguished from *T. kanvae* by the distinct spinulose conidia on the substrate. Based on the comparison of the septa and the conidial size, *T. strychnicola* (septa: 2–15, conidia: 25–50 × 5–8 μm) differs from *T. kanvae* by having 3–4 septa and conidia of 3–6 × 3–8 μm (Crous et al. 2024). The nucleotide base pair comparison between *T. strychnicola* (GZCC 24-0163, ex-type strain) and *T. kanvae* (MCC-10010, ex-type strain) revealed 63/529 bp (11.9%, 16 gaps) in ITS, 24/420 bp (5.7%, 6 gaps) in *tef*1-α, and 144/997 bp (14.4%, 4 gaps) in *rpb*2 sequence data. In addition, *Torula strychnicola* and *T. kanvae* have a close phylogenetic relationship with *T. aquatica* (KUMCC 15-0435, ex-type strain), *T. gaodangensis* (MFLUCC 17-0234, ex-type strain), *T. herbarum* (CBS 140066, ex-type strain), and *T. luguhuensis* (KUNCC 22-12427, ex-type strain) with 99% MLBS and 1.00 BIPP support (Fig. 1); base pair differences between the ex-type strain of *T. strychnicola* and these species are shown in Table 3 (Crous et al. 2015; Su et al. 2018; Hyde et al. 2017; Luan et al. 2023). Therefore, based on the morphological and phylogenetic evidence, we established *T. strychnicola* as a new species from Hainan Province, China.

**Table 3.** Comparisons of base pair differences between the ex-type strain of *Torula strychnicola* (GZCC 24-0163) and members of adjoining clades. N/A denotes unavailable sequences in GenBank.

Species	Strain no.	ITS (bp)	LSU (bp)	*tef*1-α (bp)	*rpb*2 (bp)
*T. aquatica*	KUMCC 15-0435	43/459 (9.4%, 13 gaps)	9/826 (1.1%, 1 gap)	N/A	95/796 (11.9%, without gaps)
*T. gaodangensis*	MFLUCC 17-0234	40/508 (7.7%, 12 gaps)	13/896 (1.5%, 1 gap)	N/A	N/A
*T. herbarum*	CBS 140066	39/508 (7.7%, 9 gaps)	12/891 (1.3%, 1 gap)	N/A	N/A
*T. luguhuensis*	KUNCC 22-12427	43/499 (8.6%, 15 gaps)	15/843 (1.8%, 4 gaps)	35/868 (4.0%, without gaps)	103/987 (10.4%, without gaps)

## ﻿Discussion

Through integrated morphology, culture characteristics, and phylogenetic analysis of the combined ITS, LSU, SSU, *tef*1-α, and *rpb*2 sequence data, two novel species, *Torula dispora* and *T. strychnicola*, and three previously known species, *T. chinensis*, *T. masonii*, and *T. fici*, were identified, and *T. phytolaccae* was synonymized under *T. chinensis*. All collections were reported from terrestrial habitats associated with medicinal plants in five families, including Caprifoliaceae, Liliaceae, Loganiaceae, Phytolaccaceae, and Rubiaceae, confirming that new species can be found in speciose genera such as *Torula* (Bhunjun et al. 2022). The samples of microfungi were primarily collected from dead branches, twigs, and vines, including both woody hosts, such as *Lonicera japonica*, *Gardenia jasminoides*, and *Strychnos nux-vomica*, and herbaceous hosts, such as *Disporum cantoniense* and *Phytolacca americana*. Notably, *Strychnos nux-vomica* is a highly toxic medicinal plant whose seeds contain the lethal alkaloids strychnine and brucine (Guo et al. 2018).

Morphologically, members of *Torula* share similar and overlapping features, making species delimitation based solely on morphology challenging (Su et al. 2018; Shen et al. 2021; Tian et al. 2023; Wang et al. 2023). Therefore, molecular data are crucial for interspecific identification within the genus. As concluded by Jaklitsch and Voglmayr (2016), *T. strychnicola* and *T. luguhuensis* have similar morphologies but are phylogenetically distinct species. They exhibit differences in ITS (43/499 bp, 8.6%), LSU (15/843 bp, 1.8%), *tef*1-α (35/868 bp, 4.0%), and *rpb*2 (103/987 bp, 10.4%). In addition, the taxonomic status of some *Torula* species is controversial. For example, *T. pluriseptata* (KUMCC 16-0034, ex-type strain) and *T. hollandica* (CBS 220.69, ex-type strain) were introduced as different species, but they clustered together in our multilocus phylogenetic analysis (Fig. 1). Therefore, species in *Torula* should be re-examined to verify their identity and confirm their taxonomic status, particularly if type specimens are available. Fresh collections are necessary to facilitate epitypification and provide additional data for taxonomic and molecular phylogenetic studies in *Torula*.

Bhunjun et al. (2024) argued that saprobes probably have endophytic ancestors and can change their life forms; the endophyte-to-saprobe transition hypothesis may provide a plausible evolutionary framework for *Torula* species. This does not imply extant endophytism but suggests that deep ancestral states may differ from current ecological roles. It is noteworthy that many microfungi associated with medicinal plants, particularly endophytic fungi, exhibit antibacterial or bioactive properties (Zeng 2004; Jia et al. 2016; Ali et al. 2021). For example, *Phomopsis cassiae*, an endophytic fungus from *Cassia spectabilis*, produces cadinane sesquiterpenoids that are toxic to pathogenic fungi such as *Cladosporium sphaerospermum* and *C. cladosporioides*, thereby helping the plant resist pathogenic stress (Silva et al. 2006). The endophytic *Gilmaniella* sp. from *Atractylodes lancea* produces jasmonic acid, which induces defense responses in the plant, helping it resist pathogenic fungi (Ren and Dai 2012). Similarly, some members of *Torula* have been discovered as an interesting source of secondary metabolites. For example, *Torula* sp. (YIM DT 10072) exhibited antibacterial activity against *Staphylococcus aureus* (Chunyu et al. 2018), and dehydroherbarin and o-methylherbarin have been extracted from *T. herbarum* (Narasimhachari and Gopalkrishnan 1974). These findings suggest that members of *Torula* have significant potential for bioactive properties.

In conclusion, our study described two new species and three new host records, highlighting the diversity of *Torula* species associated with medicinal plants in China.

## Supplementary Material

XML Treatment for Torula chinensis

XML Treatment for Torula dispora

XML Treatment for Torula fici

XML Treatment for Torula masonii

XML Treatment for Torula strychnicola

## ﻿Additional information

### Use of AI

No use of AI was reported.

### Author ORCIDs

Na Wu https://orcid.org/0000-0002-4837-9019

Mei-Feng Chi https://orcid.org/0009-0004-9649-5673

Hong-Zhi Du https://orcid.org/0000-0003-0350-4530

K. W. Thilini Chethana https://orcid.org/0000-0002-5816-9269

Kitiphong Khongphinitbunjong https://orcid.org/0000-0002-4548-2790

Ya-Ya Chen https://orcid.org/0000-0002-8293-168X

Kevin D. Hyde https://orcid.org/0000-0002-2191-0762

Fatimah Al-Otibi https://orcid.org/0000-0003-3629-5755

Asha J. Dissanayake https://orcid.org/0000-0002-8061-8884

Jian‐Kui Liu https://orcid.org/0000-0002-9232-228X

### Data availability

All of the data that support the findings of this study are available in the main text.
